# Multi-Ideology, Multiclass Online Extremism Dataset, and Its Evaluation Using Machine Learning

**DOI:** 10.1155/2023/4563145

**Published:** 2023-03-01

**Authors:** Mayur Gaikwad, Swati Ahirrao, Shraddha Phansalkar, Ketan Kotecha, Shalli Rani

**Affiliations:** ^1^Symbiosis Institute of Technology, Symbiosis International (Deemed University), Pune, MH 412115, India; ^2^MIT Art, Design and Technology University, Pune, MH 412201, India; ^3^Symbiosis Centre for Applied Artificial Intelligence, Symbiosis International (Deemed University), Pune, MH 412115, India; ^4^Chitkara University Institute of Engineering and Technology, Chitkara University, Rajpura, Punjab 140401, India

## Abstract

Social media platforms play a key role in fostering the outreach of extremism by influencing the views, opinions, and perceptions of people. These platforms are increasingly exploited by extremist elements for spreading propaganda, radicalizing, and recruiting youth. Hence, research on extremism detection on social media platforms is essential to curb its influence and ill effects. A study of existing literature on extremism detection reveals that it is restricted to a specific ideology, binary classification with limited insights on extremism text, and manual data validation methods to check data quality. In existing research studies, researchers have used datasets limited to a single ideology. As a result, they face serious issues such as class imbalance, limited insights with class labels, and a lack of automated data validation methods. A major contribution of this work is a balanced extremism text dataset, versatile with multiple ideologies verified by robust data validation methods for classifying extremism text into popular extremism types such as *propaganda, radicalization*, and *recruitment*. The presented extremism text dataset is a generalization of multiple ideologies such as the standard ISIS dataset, GAB White Supremacist dataset, and recent Twitter tweets on ISIS and white supremacist ideology. The dataset is analyzed to extract features for the three focused classes in extremism with TF-IDF unigram, bigrams, and trigrams features. Additionally, pretrained word2vec features are used for semantic analysis. The extracted features in the proposed dataset are evaluated using machine learning classification algorithms such as *multinomial Naïve Bayes, support vector machine, random forest*, and *XGBoost* algorithms. The best results were achieved by support vector machine using the TF-IDF unigram model confirming 0.67 F1 score. The proposed multi-ideology and multiclass dataset shows comparable performance to the existing datasets limited to single ideology and binary labels.

## 1. Introduction

Social media have become an integral part of life in the current era. People share their thoughts, beliefs, and ideas over social media platforms. Social media platforms such as Twitter, Facebook, WhatsApp, and Instagram are popular mediums of expression among people. Over 474,000 messages are posted on Twitter, and 293,000 statuses are updated on Facebook [1].

Social media platform offers extensive outreach and hence become extremely influential. This makes the social media platform a perfect tool for the extremists to spread their propaganda, radicalization, and recruitment. The extremist groups share violent messages, images, and videos over social media. The extremist organizations such as the *Islamic State of Iraq and Syria* (ISIS) [2] and *Al Qaeda* [3] use social media platforms for the spread of extremism amongst the susceptible youth.

Similarly, far-right-wing organizations such as *Alt-Right* [4] and *Proud Boys* [5] also use social media platforms to radicalize and recruit the youth. *Bill S-894* [6] claims that 73% of the violent incidents in the USA after 11 September 2001 have links with far right-wing organizations.

In the recent Christchurch mosque attack [7], perpetrators were influenced by Oslo attackers manifesto [8], spread through online means. Perpetrators live-streamed the Christchurch mosque attack on Facebook [8]. Facebook blocked the initial spread of the attack video; however, some reuploads were left undetected [9].

Online extremism research is crucial to constrain the spread of harmful ideologies amongst the susceptible youth. It also helps the regulatory bodies to monitor and control the spread of extremism.

Online extremism is carried out in the following three ways: (1) spreading propaganda, (2) attracting youths through the recruitment messages, and (3) the radical change in the perception towards an individual or community.

Propaganda is “content, generally biased, which is exploited for the personal or the political cause” [10]. Misinformation used for political gains is also termed “propaganda.” Propaganda is usually used by dictatorial administrations such as Nazism in Germany and the former Soviet Union to brainwash people. Propaganda such as “America is dead! Long Live America” [11] is used to attract people.

Jihadist propaganda mainly related to ISIS can be found in their online magazines “Dabiq” and “Rumiyah” [12]. The magazines contain propaganda in the form of glorification of the caliphate and battlefield [13]. White supremacist propaganda used by some organizations follows methods such as pamphlets similar to ISIS [11].

Radicalization is a “change in behavior, attitude, and perception towards a person or a community” [14]. Miscreants use online radicalization to mislead people by quoting their beliefs that may be political or religious [15]. Both jihadists and white supremacists use current events, encourage weapons, and violent attacks as radicalization strategies [11]. Text such as “you do realize IS wants to destroy every single nation-state, Arab or Kurd or communist does not matter, that they come across?” [16], radicalizes people in the name of religion, organization, or nation.

Recruitment in the area of extremism is the “incitement of youths to sacrifice themselves and perform violent acts on behalf of the extremist organization [17].” Jihadist-ISIS recruiters glorify ISIS fighters' death as martyrdom and exploit it as a recruitment tactic [18]. White supremacists use “feelings of inadequacy,” “anti-government themes,” and recently “coronavirus themes” to recruit disgruntled youth [11]. Extremists use posters with text such as “Join the Atomwaffen Division,” which directly calls for recruitment to the specific extremist organization [11].

Every type of extremist text and speech such as propaganda, radicalization, and recruitment has distinct features and effects. These are also explained in [19]. As social media reach is ever-expanding, extremist organizations use these platforms to spread propaganda, radicalize people, and recruit them for violent acts. Thus, it is necessary to develop a tool for identifying propaganda, radicalization, and recruitment to restrict the spread of extremism on social media platforms [16]. The online extremism research faces the following challenges:

1. Lack of publicly available datasets of the extremism text
2. Lack of the ideology-independent and balanced datasets of the extremism text
3. Lack of automated data validation methods for checking the quality of data
4. Lack of accurate automated detection methods for the online extremism text
5. Limited work on extremism content classification into categories, such as radicalization, propaganda, and recruitment

The contribution of our work is as follows:

1. Construction of multi-ideology balanced and extremism text dataset collected from multiple sources such as StormFront Dataset [20], Gab dataset [21], ISIS Kaggle dataset [22], and Twitter
2. The application of statistical data validation methods for checking the quality of the proposed dataset
3. The development of an automated framework for the detection of online extremism text, which classifies the extremism content as radicalization, propaganda, and recruitment
4. Implementation of the proposed framework with AI techniques for efficient and accurate detection of online extremism
5. Comparative performance analysis of the proposed dataset Merged ISIS-White Supremacist (MIWS) with Merged ISIS dataset (MIS), Merged White Supremacist dataset (MWS)
6. Investigation of the best feature extraction technique and classifier for the proposed extremism text dataset

This research work targets two ideologies ISIS/jihadist and white supremacist. The reason behind selecting these two ideologies is based on various factors such as infamy [23], support of violence [2, 8], and the spread of ideology online and offline [24]. Twitter is one of the most popular social media platforms with an extensive reach. Multiple studies have proved that extremists prefer Twitter for spreading propaganda, radicalization, and recruitment [16, 25, 26]. So, StormFront [20] and Gab datasets [21] are referred to as hate speech datasets. Hate speech is defined as the “attack or use of discriminatory language with reference to a person or group” [27]. At the same time, extremism can be referred to as “ideas that are opposed to society's core values which can be of various forms racial or religious supremacy or ideologies that deny basic human rights or democratic principles” [28]. There are multiple definitions of hate speech [29, 30] and similarly multiple definitions of extremism [31, 32]. However, there is a significant similarity in the definitions and interpretations of hate speech and extremism overlaps. Organizations such as the EU already consider StormFront and Gab the primary platform for right-wing extremist views [33]. Therefore, StormFront and Gab datasets are considered extremists for this paper.

## 2. Related Work

Existing literature on extremism detection is analyzed by considering the employed datasets and the classifier techniques applied.

### 2.1. Datasets

#### 2.1.1. Standard Dataset

In standard datasets, extremism text is collected, which is based on a specific ideology. The ISIS Kaggle dataset [22] was compiled by the Fifth Tribe organization to analyze the online spread of ISIS and to counteract them. The dataset contains 17,350 tweets from 112 *pro-ISIS* user accounts, collected after Paris attacks [34] in November 2015. The dataset contains 15,684 English-language tweets. This dataset includes username, location, number of followers, and timestamp of the tweet. It is used in multiple studies to detect and analyze ISIS supporters [35, 36]. The ISIS Kaggle dataset is unlabelled. Different researchers used various techniques to label the dataset. The main problem of the ISIS Kaggle dataset is that there are old accounts in the dataset, which Twitter may have suspended for discarding their hate speech policy.

The *“About ISIS Kaggle Dataset”* [37] acts as a counterpoise to the *ISIS Kaggle Dataset*. This dataset has around 122K tweets mentioning “isis,” “isil,” “daesh,” “islamic state,” “raqqa,” and “mosul.” The dataset is unlabelled, containing pro-ISIS accounts, as the data collected is based on keywords. Most of the accounts are unavailable or deleted in the *ISIS Kaggle dataset*.

In *ISIS Religious Text Kaggle dataset* [38], data is collected by Fifth Tribe. This dataset is compiled by scraping of fifteen and nine issues of Dabiq and Rumiyah magazines, respectively. The dataset contains a total of 2,685 texts. Standard datasets related to jihadism or ISIS ideology are unlabelled and contain suspended accounts.

There are very few standard datasets available in the literature on White supremism hate speech. de Gibert et al. [20] collected the extremist hate speech data from StormFront and the White supremacist website. de Gibert et al. compiles 10,568 posts and manually annotates them as *hate*, *nohate*, *relation,* and *skip*. The experts identified a total of 1,119 hate posts and 8,537 nohate posts. de Gibert et al. compare the characteristics of the StormFront dataset with the Hatebase dataset. The StormFront dataset has a major issue of class imbalance.

Kennedy [21] collected 27,000 posts from the Gab social network. Gab social network claims to preserve the freedom of speech and has become a haven for disseminating hate speech. The authors categorize posts into attack on human dignity (HD), call for violence (CV), and offensive/vulgar language (VO). The authors further classify HD and CV into implicit, explicit, race/ethnicity, nationality, gender, religion, sexual orientation, ideology, political ideology, and mental/physical health. The authors considered three classes, HD, VO, and hate (a combination of HD and CV), for the classification.

The standard datasets in both ISIS and White supremacist ideology are very few. The accounts from which data is collected may have been inactive, suspended, or deleted by the user or the social media platforms. Therefore, the labels provided within datasets are inadequate to provide insights into extremism linguistics in both ideologies. Furthermore, there is a lack of data validation techniques to evaluate the standard datasets. Hence, many researchers prefer to collect extremism-related data from various sources, and manual annotation is performed due to these issues.

#### 2.1.2. Custom Dataset

Similar to standard datasets, custom datasets are created to represent specific ideologies. Berger [25] in 2014 collected 20,000 ISIS-related accounts from Twitter. The author analyzed the location of supporters, languages spoken by the supporters, identification information of supporters, when the supporter accounts were created, the content of posts by ISIS supporters, and the methods used for the identification of propaganda and recruitment.

Chatfield et al. [16] collected 3,036 tweets from @shamiwitness, who was a known ISIS sympathizer. The tweets of @shamiwitness were manually annotated with propaganda, radicalization, and recruitment by the authors. The account of @shamiwitness is now suspended so that no further analysis can be performed. The authors rely on manual data validation methods with no statistical evidence.

Rowe and Saif [39] used the dataset provided by O'Callaghan et al. [40] as the SEED dataset. From the SEED dataset, the authors identified 154K users suspected of spreading ISIS propaganda. The authors collected 3,200 tweets from each user resulting in 104 million tweets. The authors found 43% of tweets in English, 41% in Arabic, and the rest in Spanish and Dutch. For validation of the dataset, the authors used *interrater agreement* using two annotators. In addition, the authors used a sample of 2,000 tweets for manual validation, and the agreement of annotators was between 0.4 and 0.6 Fleiss' Kappa. The authors did not use any other statistical technique for data validation.

Kaati et al. [41] used 66 Twitter users as seeds obtained from Shumukh al-Islam Forum. The authors used hashtags such as #ISLAMICSTATE, #ILoveISIS, and #AllEyesOnISIS. Thus, a total of 27,253 English pro-ISIS tweets and 16,000 Arabic pro-ISIS tweets were collected. The authors did not provide any information on data validation.

Ashcroft et al. [42] used similar methods described by Kaati et al. [41] to collect a total of 7,500 tweets consisting of pro-ISIS, anti-ISIS, and random contexts. Unfortunately, most of the data were collected from older accounts, which may have been suspended.

Benigni et al. [43] used a two-step snowballing process to collect accounts related to ISIS. In the first step, the authors used five seed accounts to collect 1,345 unique accounts. The authors collected 1,19,156 user accounts in the second step, which followed or related to 1,345 accounts of the previous step. Thus, the authors collected a total of 862M tweets by the end of step two. Unfortunately, due to the Twitter data-sharing policy, the tweets collected by the authors were not available to the public.

Abrar et al. [44] gathered 13,369 terrorism-supporting tweets, 16,506 terrorism-nonsupporting tweets, and 38,617 random tweets. However, the authors neither mentioned any seed accounts or terrorism-specific keywords used to gather tweets nor performed any data validation methods on the collected dataset.

Ahmad et al. [45] gathered ISIS-related tweets using keywords such as ISIS, bomb, and suicide. The authors also used manually identified seed words for identifying ISIS-related tweets. The authors conclude that 12,754 tweets were extremists and 8,432 were nonextremists. However, the research work lacks data validation on the collected data.

Asif et al. [46] used the Facebook pages of news agencies such as PTV news, Dawn, and Geo to gather extremist texts. A total of 19,497 posts were collected, from which 5,279 were labeled as moderate, 6,912 as highly extreme, 2,991 as low extreme, and 4,315 as neutral. The authors used survey-based validation, using 109 random people. However, the authors used only a sample of 25 posts which may not represent the whole data.

Gialampoukidis et al. [47] collected ISIS-related data by searching five keywords provided by law enforcement agencies and domain experts. So, this resulted in 9,528 tweets from 4,400 suspected ISIS-supporting users. Unfortunately, this dataset is unavailable due to the data-sharing policy of Twitter.

The researchers collected data for extreme right-wing, White supremacist ideology from different sources and locations. Jaki and De Smedt [48] collected 50,000 tweets from about 100 Twitter users suspected of supporting far-right ideology in Germany. The authors also collected 50,000 neutral tweets. The authors did not provide any details about data validation methods.

Berger [26] manually collected data from 41 Twitter users who supported the alt-right movement. By checking these accounts' followers, the author collected 27,895 user accounts suspected of supporting the alt-right movement. Berger also collected data from 33,766 neutral user accounts. The author used manual validation for the collected data. *Alt-Right Demographics dataset* is not available publicly due to Twitter data sharing policies. So, the reproducibility of results is not possible.

Some researchers also collected data from multiple ideologies. For example, De Smedt [49] used a multidomain perspective for extremism detection. The authors divided the text into jihadism (ISIS), extremism (far right-wing from Germany, Belgium, Netherlands, US, UK, and Canada), sexism, and racism. The authors collected 50,000 tweets for jihadism, 92,500 tweets for extremism, 10,000 tweets with 15,000 Facebook posts for racism, and 65,000 posts from Incels.me about sexism. The authors used *hate* and *safe* labels for extremism, jihadism, sexism, and racism domains. The authors also used left and right labels for the extremism domain. The authors also analyzed demographic profiling, psychological profiling, sentiment analysis, and network analysis with detection. Unfortunately, De Smedt et al. do not provide access to the datasets due to strict Twitter policies on data sharing.

Similarly, Berger [23] compared two ideologies ISIS and Nazis, by collecting data from Twitter. First, to identify the users with White supremacist and Nazi sympathies, the author used 18 seed accounts. The author then collected around 200 tweets from a total of 25,406 followers of these 18 seed accounts. Then, for analysis, the authors used 4,000 highly relevant Nazi-sympathizing accounts. Finally, the author used a similar strategy to collect 4,000 ISIS sympathizing accounts from Twitter.

Heidarysafa et al. [50] compared the women-specific content of ISIS with women-specific Catholic preaching. The authors collected 20 articles from Dabiq and Rumiyah targeting women and 132 articles from catholicwomensforum.org. The authors relied on manual validation but did not provide any statistical evidence.

Araque and Iglesias [51] used different datasets such as Pro-Neu, Pro-Anti, Magazines, SemEval2019 [52], and Davidson [53] to classify radicalization and hate speech using AffectiveSpace and SenticNet. The authors also used multiple features such as TF-IDF and similarity-based sentiment projection (SIMON) for prediction.

Mussiraliyeva et al. [54] collected religious extremist posts from VKontakte [55] social media platforms in the Kazakh language. The authors used different extremist keywords such as “kafir” and “kill” to identify extremist texts. The annotation of an extremist text is based on the appearance or absence of selected extremist keywords within the text.

From Table 1, it is observed that issues plaguing custom datasets are *data availability*, *result reproducibility*, *binary classification*, *data imbalance,* and *single ideology focus*. Data availability is an issue due to the policy of social media. So, in turn, this affects the reproducibility of the results for other researchers. Nearly all the researchers using the custom datasets use binary classification, which is inadequate for deeper analysis. The extremism data are less than nonextremist data. Thus, the class imbalance is inherent in the custom datasets. The biggest problem of both standard and custom datasets is that their focus is on a single ideology.

Thus, there is a need for a generic dataset of the extremism text, which accounts for multiple ideologies. Additionally, the dataset should help classify extremism text into popular types, that is, propaganda, radicalization, and recruitment. Thus, a generic dataset with multiple ideologies and a single-model multiclassification can efficiently detect online extremism text. These challenges are further explained in Section 3.

### 2.2. Challenges with Existing Online Extremism Datasets

**Figure 1:**
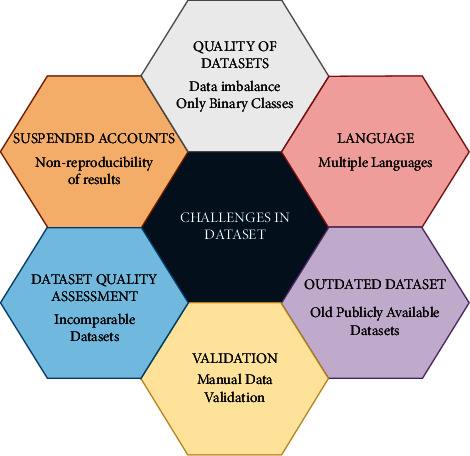
Challenges in dataset.

There are various research gaps found in the dataset of online extremism text. The following challenges are observed in online extremism text datasets as illustrated in Figure 1:

#### 2.2.1. Data Imbalance and Binary Classification

Data imbalance is a serious problem for online extremism datasets. StormFront dataset [20] and Gab dataset [21] are good examples of class imbalance. As extremism data is the fraction of the total data on social media, creating a balanced class dataset is challenging.

Another problem with the dataset is binary or at the most three-class classification of extremism data. Extremist-nonextremist, pro-ISIS-not Pro-ISIS, and hate-not hate are some of the available binary classes. The third class, if available, is either called “irrelevant” or “neutral.” Unfortunately, this classification does not provide analytical insights into the extremism text. Thus, limiting the understanding of extremist activities on social media. Moreover, the expressions of extremism are complex and change over time. Therefore, it is necessary to create the categories based on the context of extremist texts.

#### 2.2.2. Language

The extremism in different ideologies is spread through different languages. Thus, the identification of the extremist text becomes more challenging. Most researchers use English as the global language. The extremist widely uses English to spread their ideology worldwide. Multiple studies by Jaki and De Smedt [48], and De Smedt [49], have addressed online extremism in Dutch and German languages. Rowe and Saif [39] collected dataset containing ISIS-related tweets in English, Arabic, Spanish, and Dutch languages, but limited their research studies to English and Arabic languages.

#### 2.2.3. Outdated Dataset

Standard datasets such as ISIS Religious Text dataset [38] are old. This is because these datasets were obtained during the early days of ISIS. Another issue is the strict data-sharing policy of social media, which makes updating old datasets impossible. This strict data-sharing policy is also one reason for the fewer numbers of standard datasets.

#### 2.2.4. Validation

Most researchers use manual validation with the interrater agreement. As it is impossible to validate an entire data manually, few random samples are used for data validation. Thus, bias is introduced unknowingly. The number of experts also affects the bias in data validation. Fewer experts may give good interrater agreement, but the bias persists. The use of multiple experts may lower the bias, but the *interrater agreement* may deteriorate [46].

#### 2.2.5. Data Quality Assessment

In online extremism research, researchers often collect their own data [26, 35]. Due to the restriction of social media and other issues, previous custom datasets are not available publicly. So, the comparison of datasets is a huge issue in online extremism research. This also leads to another problem of comparison of results. As no study uses the same dataset, comparing results with different methods and techniques is difficult in online extremism detection research.

#### 2.2.6. Suspended Accounts

Social media has a strict policy on violence and hate speech [29, 56]. Thus, many accounts with such extreme ideologies get suspended immediately. So even after data collection, other researchers cannot reproduce the results due to the unavailability of suspended accounts.

This work aims to address *data quality challenges*, *data validation*, *data imbalance*, and *binary classification* in extremism datasets. The challenges about languages and suspended accounts do not fall into the scope of this work.

### 2.3. Classifiers

Network-based, machine learning-based, and deep learning-based techniques are popularly used in online extremism research [19].

#### 2.3.1. Network/Graph-Based Techiques

Network/graph-based techniques are preliminarily used due to the following reasons:

- To cluster extremists on social media
- To identify extremist communities on social media
- To perform data collection by identifying connections among the extremists

Since 2015, only few studies use the network/graph-based approach. Agarwal and Sureka [57] used the *breadth-first search* and *shark search algorithms* to find the extremists and their communities on YouTube. The authors used the class name relevant (extremist) and irrelevant (nonextremist). By using the shark search algorithm, the authors achieved an accuracy of 0.74 and an F1 score of 0.85.

Saif et al. [58] used closegraph to extract subgraphs of extremists on Twitter. The authors used these subgraphs as features for machine learning algorithms such as Naïve Bayes, maximum entropy, and SVM. In addition to subgraphs, the authors used unigram, sentiment, and semantic features. The authors concluded that SVM performs the best with a precision, recall, and F1 score of 0.93 for pro-ISIS and anti-ISIS classes.

Petrovskiy and Chikunov [59] also used graph techniques to extract features such as node page rank, hub and authority measure, and betweenness centrality. These features are then used as input for algorithms such as logistic regression, random forest, and XGBoost. The XGBoost algorithm outperforms other algorithms with a ROC curve of 0.95 for train and 0.94 for test data.

Moussaoui et al. [60] used a possibilistic graph for extremist community detection. Features such as semantic similarity, structural similarity, and possibilistic similarity are extracted using a possibilistic graph-based approach. The authors used subgraphs as features input to machine learning algorithms. The authors used *Naïve Bayes*, *multinomial Naïve Bayes* (MNB), and *stochastic gradient decent* (SGD) classifiers for extremism detection. SGD achieved a precision of 0.81 and an accuracy of 0.86 for extremism detection.

Network/graph techniques are used mostly to identify communications and interconnections but suffer from multiple challenges:

- It cannot work for disconnected nodes in the graph
- Semantic analysis of extremism text cannot be performed with network/graph techniques

Thus, to overcome the network/graph approach challenges, machine learning-based and deep learning-based methods are used for online extremism detection.

Machine learning-based approach is used for the classification of data into extremist, nonextremist, or neutral [46, 61] or the classification of data into extremist and antiextremist [39, 42].

#### 2.3.2. Machine Learning-Based Techniques

In machine learning-based approach different classifiers such as MNB [46], *logistic regression* [65], SVM [46], *random forest* [68], and *XGBoost* [71] are used for online extremism detection.

Agarwal and Sureka [64] used k-nearest neighbor and libSVM to identify hate-oriented text from Twitter. The authors used the term frequency as the feature. The authors got an accuracy of 0.97, a precision of 0.78, and a recall of 0.83.

Asif et al. [46] used MNB and *support vector classifier* (SVC) to classify Facebook posts and comments as *moderate*, *high extreme*, *low extreme,* and *random*. SVC performs better for the classification than multinomial Naïve Bayes, giving an accuracy of 0.82.

Benigni et al. [43] proposed *iterative vertex clustering and classification* (IVCC) for extremism detection. The authors also used *k-means*, *Louvain grouping*, and *Newman method* for extremism detection. The authors classify Twitter users into ISIS members, nonmembers, and suspended. IVCC outperforms other classification methods with an accuracy of 0.96 and an F1 score of 0.93.

Araque and Iglesias [36] used feature engineering by creating emotion features (EmoFeat) and similarity-based feature extraction (SIMON) methods. The authors labeled the data as positive (extremist) and negative. The authors got the highest F1-score of 0.94 for EmoFeat and SIMON, with the dataset containing extremist and neutral tweets.

Ashcroft et al. [42] used a *stylometric*, *sentiment,* and *time-based* feature for online extremism detection. The authors classify data into radical and nonradical. The authors used SVM, Naïve Bayes, and AdaBoost. AdaBoost gave a precision of 0.88, specificity of 0.99, and sensitivity of 0.79, with all the features outperforming other algorithms.

Fernandez et al. [35] divided extremists into individual (*micro*) influence, group (*meso*) influence, and global (*macro*) influence based on their tweets. The authors used the *collaborative filtering* and *Naïve Bayes* classification method. The authors used precision as a performance metric. Using Naïve Bayes, the precision obtained for micro is 0.79, for meso is 0.69, and for macro is 0.90.

Mussiraliyeva et al. [62] divided Kazakh language posts from VKontakte [55] into extremist and nonextremist classes. The authors used different classifiers such as logistic gegression, MNB, and SVM. The authors also used decision tree-based classifiers such as random forest and gradient boosting. From all these classifiers, gradient boosting with word2vec gave the best F1 score of 0.86.

Mussiraliyeva et al. [54] used multiple features such as linguistic inquiry and word count (LIWC), part-of-speech (POS), and TF-IDF. The authors used numerous machine learning algorithms such as SVM, k-nearest neighbors (KNN), decision tree, random forest, Naïve Bayes, and logistic regression. The KNN using the oversampling method with statistical and TF-IDF features gives an accuracy of 0.99 for religious extremism classification.

Araque and Iglesias [51] used a combination of multiple features such as AffectiveSpace, SenticNet, TF-IDF, and SIMON. The authors used machine learning algorithms such as logistic regression and linear SVM.

De Smedt et al. [67] identified extremist hate speech within English, Arabic, and French language tweets. The authors used character trigrams as features. The tweets were labeled as hate and safe. The authors used libSVM as the classifier. The F1 score for the English language was 79, for French was 80, and for Arabic was 84.

Ul Rehman et al. [63] used *religious words*, *radical words,* and *bad words* to detect online extremism. The authors used two classes, extremist and nonextremists. The authors preferred different algorithms such as *Naïve Bayes*, SVM, and *random forest* for the classification. The SVM with all the features outperforms other algorithms with an F1 score of 0.87.

Sharif [61] divided tweets into *pro-Taliban*, *pro-Afghan*, *neutral*, and *irrelevant*. The authors used unigrams, bigrams, and TF-IDF for feature extraction. The authors also used principal component analysis (PCA) to reduce dimensions. The research work used *Naïve Bayes*, SVM, and *random forest*. SVM with TF-IDF and bigrams offers the best precision of 0.84.  Table 2 provides a comparison of all these studies in brief.

#### 2.3.3. Deep Learning-Based Techniques

Even if machine learning-based approaches are popular, they face some challenges such as the following:

- They depend heavily on manual feature extraction or feature engineering
- Not suitable for large and unstructured datasets
- Context identification is a challenge

These issues of machine learning methods can be addressed by using the deep learning approach. In the deep learning-based approach, the researchers have tried CNN [45], gated recurrent unit (GRU) [45], LSTM [65], and BERT [65].

A deep learning-based approach is used due to the following reasons:

- Automated feature extraction
- Pretrained models on a large corpus

Recently deep learning approaches are routinely used in online extremism detection due to automated feature extraction and large computing power.

Kaur et al. [72] classified data into *radical, nonradical*, and *irrelevant* classes. The authors used word2vec for features extraction. Multiple algorithms such as SVM, *maximum entropy,* and *random forest* were used. The authors primarily focused on the deep learning approach using LSTM. LSTM with word2vec gives the best precision of 85.96.

Ahmad et al. [45] used n-grams, TF-IDF, and bag-of-words (BoW) as feature extraction methods for online extremism detection. The authors used the CNN model, LSTM model, FastText with word embedding, and GRU. The LSTM with CNN model offers an accuracy of 0.92 and a precision of 0.90 outperforming other algorithms.

Alatawi et al. [65] used BERT to detect hate speech related to White supremism on Twitter. The work used pretrained networks such as *Google News Word Vectors*, GloVe trained on Wikipedia, and GloVe trained on Twitter. The authors also train the extremist data using word2vec, referring to it as White supremacist word2Vec (WSW2V). BERT with WSW2V outperformed other techniques with an F1 score of 0.79 and a precision of 0.80. The direct comparison between approaches in online extremism detection is a problem. This is due to the use of different datasets, most of which are custom and not publicly available.

Mussiraliyeva et al. [73] in a recent study used CNN and LSTM to classify extremist posts collected from VKontakte. The CNN and LSTM both provide an AUC of 0.99 for extremism classification in the Kazakh language.  Table 3 compares the studies employing deep learning for extremism detection.

### 2.4. Proposed Architecture

**Figure 2:**
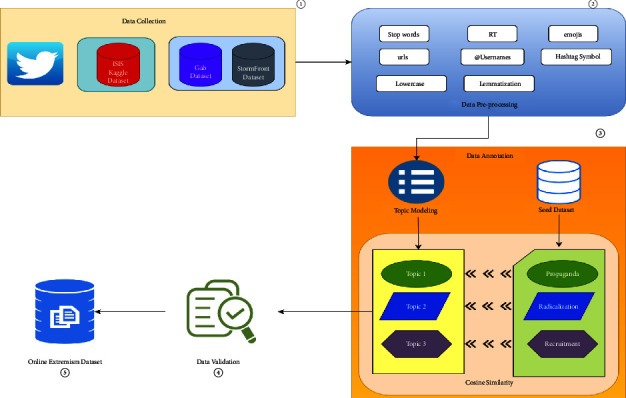
Proposed architecture.

This section proposes the architecture for constructing the dataset, which will be used to classify extremism text into propaganda class, radicalization class, and recruitment class, with discussions on data validation methods. The architecture is modularized into the following phases: data collection, data preprocessing, data annotation, and data validation which are shown in Figure 2.

#### 2.4.1. Data Collection

The construction of the proposed dataset was performed by collecting data from popular standard extremist text datasets and recent extremist tweets collected from Twitter.

#### 2.4.2. Standard Dataset

In this phase, three different datasets were chosen, namely, ISIS Kaggle dataset (∼15,000), StormFront dataset by de Gibert et al. (∼1100), and Gab Hate Corpus by Kennedy et al. (∼8000). Initially, these datasets were divided according to ideology, ISIS dataset as jihadist, while StormFront and Gab datasets as White supremacist. All these three datasets together contain around 24,900 extremist tweets. StormFront and Gab have two unique labels as hate and nonhate labels, while ISIS contains only extremist tweets. In addition, the StormFront dataset accounted for the posts between the years 2002 and 2017, while no data collection timeline is given for Gab dataset. Twitter was the preferred social media platform for collecting extremist tweets as it is the first choice for the extremists to reach out to the target audience. In addition, it is popularly used in research work [48, 67] due to its easy accessibility and microblogging format.

#### 2.4.3. Data Extraction from Twitter

As the standard dataset has its challenges such as outdated text, as mentioned in the previous section, we collected recent extremism tweets from Twitter from January 2021 to June 2021.

Twitter API allows the collection of real-time tweets with different parameters. Twitter API provides a choice to collect tweets based on specific terms or hashtags, tweets of a specific user, tweets from a specific geographical area, and tweets of a specific language. Twitter APIs also give additional information such as username, location, and @user mentions in the tweet. Different queries were formulated, and the final query was selected as

(1) $$\begin{array}{c} Query\left[\right]=Search\left\{{search}_{term},time\right\}\text{.} \end{array}$$

To collect ISIS extremism text, specific keywords such as “*murtadeen*,” “*munafiqeen*,” “*khawarij*,” “*tafkir*,” “*kuffar*,” and “*murtad*” were used. These are popularly used ISIS-related words obtained from works such as [16, 41]. In addition, the keywords such as “*white genocide*,” “*white lives matter*,” “*it's okay to be white*,” and “*anti-white*” were used to collect White supremacist-related tweets. These White supremacist supporting keywords were obtained from [74–76].

**Figure 3:**
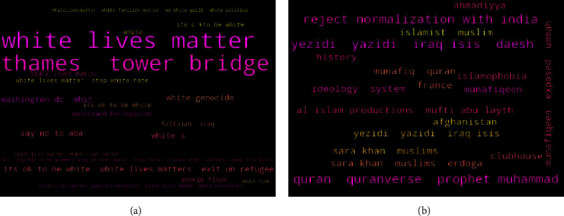
Word cloud of hashtags for data collected for (a) White supremacists and (b) jihadist-ISIS with Twitter search terms.

A total of 2,000 ISIS supporters and 2,000 White supremacist supporting tweets were collected. All these collected tweets are in the English language.  Figure 3 provides keywords used and the wordcloud of hashtags found for White supremacist and jihadist-ISIS supporting tweets.

#### 2.4.4. SEED Dataset

One of these works aims to detect extremism and classify text into propaganda, radicalization, and recruitment. To achieve this, we collect examples of propaganda, radicalization, and recruitment from the existing literature. The collected examples are from both ideologies, jihadist-ISIS, and White supremacist.

Most of the examples from the literature [39, 46, 65] were manually annotated with fewer experts and are subject to bias. Hence, we extract examples from multiple resources [11, 16]. The assumption is that the seed example from different sources provided by different experts may reduce expert bias. A total of 100 examples were identified for jihadist-ISIS and 100 examples of White supremacists on propaganda, radicalization, and recruitment.

As the examples are taken from different research works, they have multiple keywords and different contexts associated with them, reducing the overall bias of the SEED dataset. In Table 4, a few examples are presented to show the tweets and posts considered propaganda, radicalization, and recruitment by respective studies.

#### 2.4.5. Data Preprocessing

In this phase, data preprocessing is carried out in the following steps:

- *Removing Stopwords*. Stopwords were removed at this step. Then, the words representing nouns, verbs, adverbs, and adjectives were selected. This ensured the inclusion of only relevant words in the final process
- *Removal of URLs*. URLs were removed. Some studies do use URLs for further analysis. However, with standard datasets, many URLs are obsolete. Hence, the inclusion of URLs is not considered in this study
- *Removal of Emojis, Hashtag Symbols, Retweet Symbols (RT), And Digits*. Hashtag symbols and RT symbols are not the focus of this study. Numbers and digits may interfere with word analysis, hence are excluded
- *Removal of @username Mentions*. Due to constant communication between users, mention of usernames is fairly common. This may help algorithms to construct the pattern with usernames to build linkage
- *Lowercase*. All words are converted to lowercase so that case of the alphabet does not affect the prediction results
- *Lemmatization*. Lemmatization of texts is also performed so that pronouns and the tense of words may not affect the final prediction

**Figure 4:**
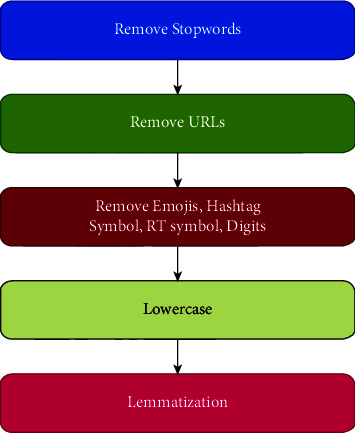
Data preprocessing.

The preprocessing steps are illustrated in Figure 4.

### 2.5. Data Labelling

#### 2.5.1. Topic Modelling

Topic modelling is a method to recognize, understand, and summarize a large collection of textual information. Topic modeling is a way to extract a group of words (topics) that accurately represent the collection of documents in a corpus. It is also a form of text mining in which word patterns in a corpus are identified.

### 2.6. Latent Dirichlet Allocation (LDA)

LDA is a probabilistic topic modeling algorithm, which extracts topics from documents, and words in the document are collected by observing their probabilistic distribution.

There are different techniques other than LDA to identify abstract information from a corpus. *Latent semantic analysis* (LSA) [78] and *probabilistic latent semantic indexing* (pLSI) [79] are some of them.

LDA focuses on topic identification and analysis, while LSA focuses on reducing matrix dimensions. LSA converges faster due to dimensionality reduction but at the expense of accuracy. pLSI uses a probabilistic model with dimensionality reduction and is faster with acceptable accuracy. Top2Vec is a recent development in finding topics within the documents. Top2Vec [80] has considerable advantages over LDA such as no need for stopword removal, stemming, or lemmatization. BERTopic [81] too has advantages such as deep learning and visualization. But both Top2Vec and BERTopic require a good amount of data which is a limitation of our study. In addition, LDA is preferred as we need a specific number of topics. Moreover, LDA is used in multiple studies for extremism detection, thus making LDA reliable for extremism detection research.

**Figure 5:**
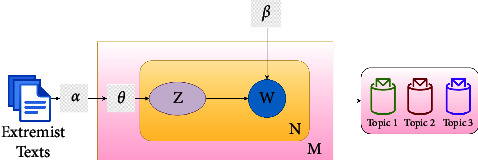
LDA model.

LDA assumes the mixture of the probabilistic distribution of topics over corpus and words over the topic. LDA works in the following ways as shown in Figure 5:

(i) Assume there are *k* topics over the entire corpus
(ii) Distribute *k* topics across document *M* which is per-document topic distribution also denoted as *α*. The topic distribution for document *M* is denoted as *θ*
(iii) Calculate *z* which is the topic of *n*^th^ word in document *M*, while *N* is the number of words in the given document
(iv) Calculate the probability of word *w*w which belongs to a particular topic based on the following:
  - Unique topics in document *M*.
  - The frequency of the word *w*w that has been assigned to a particular topic across all documents is also denoted as *β*.

For this study, it is needed to identify different topics within the extremism corpus. Later, these topics are compared for the labeling of extremist texts. So, LDA is used to extract topics from the extremism corpus due to its advantages as mentioned above and as described in Figure 5.

#### 2.6.1. Cosine Similarity

Cosine similarity computes the similarity between vectors. It calculates the cosine of the angle between vectors and determines whether vectors point in the same direction. In NLP, cosine similarity is commonly used to measure the similarity between the extracted features. Cosine similarity takes a total length of vectors; for example, considers TF-IDF vectors, thus considering repetitions of the word [82]. This property is used to identify unique words for a particular class in this work. So, cosine similarity is considered for assigning labels from SEED datasets to primary datasets.

**Figure 6:**
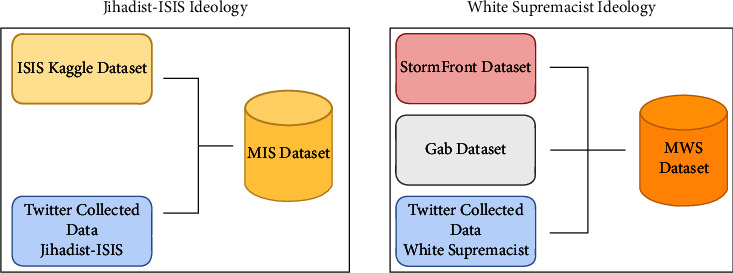
Datasets and their combinations.

In this work, data labeling is designed to be a four-step process and the steps are described as follows:

*(1) Step 1*. In the first step, datasets are merged according to ideology. The ISIS Kaggle dataset was merged with recent tweets of jihadist-ISIS collected from Twitter, referred to as the Merged ISIS dataset (MIS). Similarly, StormFront dataset, Gab dataset, and White supremacist tweets collected from Twitter merged to form Merged White Supremacist dataset (MWS). This process is shown in Figure 6. Only the text or tweet data is selected from these standard datasets, everything else is discarded. To preserve the distinct characteristics of ideology, we adopt the strategy to identify individual clusters within the ideological datasets. To identify these clusters, the topic modelling approach was chosen [83]. For feature extraction, TF-IDF is used. TF-IDF calculates important words in the corpus concerning documents. However, even if TF-IDF presents important words, it lacks in identifying context. So, to extract topics from the primary dataset, latent dirichlet allocation (LDA) [83] is used. This work aims to classify text into three classes: *propaganda*, *radicalization,* and *recruitment*; three topics are extracted from the MIS and MWS datasets. To achieve this, GridSearchCV [84] is applied to the LDA model with hyperparameters such as n_topics = [3–5], learning_rate = [0.999, 0.99999], cv = 10, and batch = “online.” Using these hyperparameters, the model with the best results gives n_topics of 3 with distinct words per topic.

**Figure 7:**
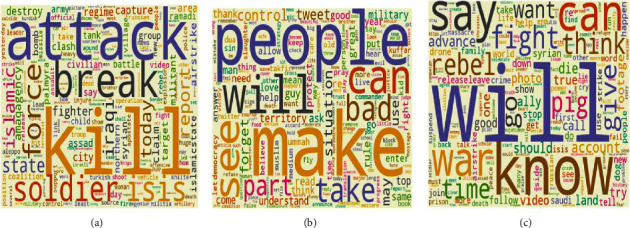
Word clouds for (a) topic 0, (b) topic 1, and (c) topic 2 in MIS dataset.

**Figure 8:**
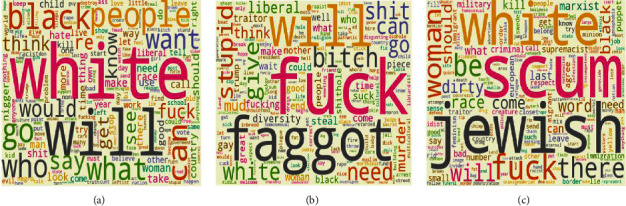
Word cloud for (a) topic 0, (b) topic 1, and (c) topic 2 in MWS dataset.

**Figure 9:**
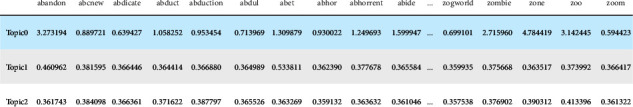
LDA ranking of jihadist-ISIS words for three topic.

**Figure 10:**
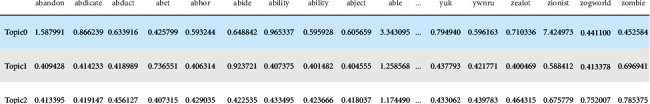
LDA ranking of White supremacist words for three topics.

*(2) Step 2*. In the second step, we extract a single topic for propaganda, radicalization, and recruitment examples for each SEED dataset of jihadist-ISIS and White supremacist ideology using LDA. This results in a single topic with respective important words in propaganda, radicalization, and recruitment. Figures 7(a)–7(c) show the word clouds of three topics obtained from the MIS dataset. Similarly, Figures 8(a)–8(c) show word clouds of the three topics obtained from the MWS dataset. These word clouds are based on the topic score obtained using LDA for MIS and MWS datasets, as shown in Figures 9 and 10.

**Figure 11:**
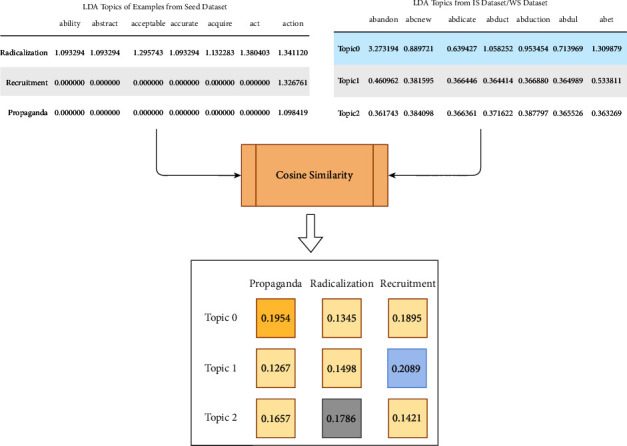
Data labeling.

*(3) Step 3*. To label text in the IS dataset and the WS dataset, cosine similarity [85] between the topics of individual MIS and MWS datasets, with the topic of propaganda, radicalization, and recruitment from SEED dataset, is calculated. This results in similarity matrix. When similarity is maximum for topic and label, the respective label, propaganda, radicalization, and recruitment, is assigned to a particular topic. Thus, documents in IS and WS datasets with the topics labeled are propaganda, radicalization, and recruitment.  Figure 11 shows the complete process of data labeling. The calculated cosine similarity between seed labels and identified topics is small. There are different reasons for low cosine similarity, such as few seed examples, and not enough significant features in SEED dataset. This low cosine similarities are accepted as two different datasets i.e., SEED dataset and tweet + website dataset are compared.

**Figure 12:**
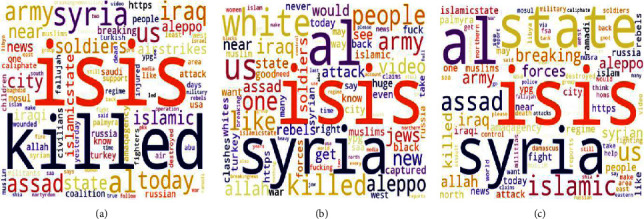
Word clouds for (a) propaganda, (b) radicalization, and (c) recruitment class from MIWS.

This research work aims to develop an ideology independent extremism detection model. So, to achieve this aim, two datasets MIS and MWS datasets, are merged. This is carried out by retaining tweets or posts, topics, ideology, and labels from both datasets. This merged dataset will be henceforth referred to as **Merged ISIS-White Supremacist dataset** (**MIWS**). As seen in Table 5, for the MIS dataset, topic 0 is labeled as propaganda, topic 1 as radicalization, and topic 2 as recruitment, as significant cosine similarity was found with the respective classes in the SEED ISIS dataset. On the other hand, in the MWS dataset, topic 0, topic 1, and topic 2 are labeled as radicalization, recruitment, and propaganda as a significant similarity score was found with respective classes of the SEED White Supremacist dataset. Figures 12(a)–12(c) can provide important words in the MIWS dataset for propaganda, radicalization, and recruitment.

### 2.7. Data Validation (MIWS)

In this Section, we discuss the statistical tests, which will be employed for the data quality assessment. We employed three statistical techniques that are *cosine similarity*, *Wilcoxon signed-rank test*, and *chi-square test*.

#### 2.7.1. Cosine Similarity

Cosine similarity can be used to compare the similarity between samples. Propaganda, radicalization, and recruitment are compared based on words and their TF-IDF score. The cosine function was applied to a pair of classes. These pairs are described in Table 6. Thus, each class is represented by distinct unique words, and they influence each class differently.  Figure 11 shows cosine similarity between different datasets, while in Table 6, similarities are seen within classes of the same dataset. Thus, even if values in Table 6 look significant, there is not enough similarity within the dataset given the N1 and N2 sizes.

#### 2.7.2. Wilcoxon Signed-Rank Test

Wilcoxon signed-rank test [86] is a nonparametric test. It can determine whether the two samples are collected from the population of the same distribution. Wilcoxon signed-rank test is also used to compare two closely related samples and perfectly matched samples.

**Figure 13:**
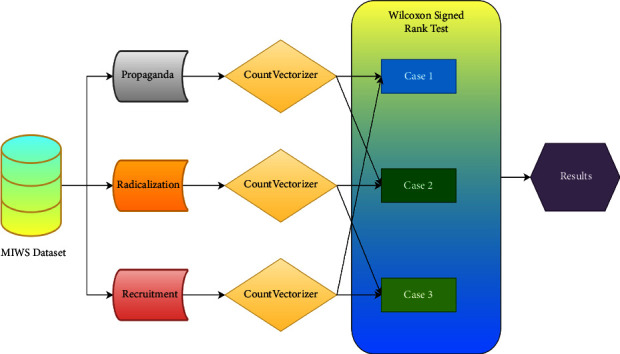
Complete process for performing Wilcoxon signed-rank test.

In this paper, Wilcoxon signed-rank test is used to prove whether the selected random samples belonged to a particular class, i.e., propaganda, radicalization, or recruitment.  Figure 13 shows detailed experiments performed to calculate the Wilcoxon signed-rank test. CountVectorizer [87] was applied for feature extraction to the corpus of each class separately. CountVectorizer returns the matrix with the count of tokens. This was performed so that higher count words from each corpus may get priority. TfidfVectorizer [88] was also considered for this experiment but leads to a dimensional mismatch for Wilcoxon signed-rank test. The TfidfVectorizer also produces *p* values >0.05 when dimensions are matched.

To perform these experiments, a null hypothesis is required, which is as follows:

  H0-medians of word count of classes are equal. Therefore, there is no significant difference between classes
  H1-medians of word count of classes are not equal. Therefore, there is a significant difference between the classes

Wilcoxon signed-rank test compares examples based on two test statistics. First, *W* test statistics which is the sum of ranks with differences below or above zero. The second is the p value which is the confirmation against the null hypothesis. Together, *W* and p value determine the validity of the null hypothesis.

To calculate *W*, the following procedure is performed:

Let *N* be the sample size, and for pairs, let *x*_1,*i*,_ and *x*_2,*i*_ denote the measurements.

(i) Calculate |*x*_2,*i*_–*x*_1,*i*_| and sgn (*x*_2,*i*_–*x*_1,*i*_), where sgn is the sign function that returns the sign of a real number
(ii) Exclude the pair with |*x*_2,*i*_–*x*_1,*i*_| = 0, and the new sample will be *N*_*r*_
(iii) Order the remaining pair in an ascending order with a difference of |*x*_2,*i*_–*x*_1,*i*_|
(iv) Rank the pairs with the smallest nonzero difference as 1. Let *R*_*i*_ denote the rank
(v) The test statistic *W* is calculated as
  (2) $$\begin{array}{c} W=\overset{N_{r}}{\underset{i=1}{\sum }}\left[sgn\left(x_{2,i}-x_{1,i}\right)∙R_{i}\right]\text{.} \end{array}$$

The p value is considered as the evidence against the null hypothesis. The null hypothesis is rejected if the p value is <0.05. This threshold of 0.05 or 5% is considered a level of significance. The count for each word representing classes is calculated.

As the classification is a multiclass classification, the tests are divided into different cases which are as follows:

- Case 1: here, the propaganda class and recruitment class are compared using CountVectorizer of *n* number of words from both classes
- Case 2: here, radicalization class and propaganda class are compared using CountVectorizer of *n* number of words from both classes
- Case 3: here, recruitment class and radicalization class are compared using CountVectorizer of *n* number of words from both classes

Table 7 shows cases, their samples, test statistics, hypothesis, and inference. The Wilcoxon signed-rank test provides test statistic “*W*” which is used to calculate the p value from the reference table [86].

#### 2.7.3. Chi-Square Test

The chi-square test is a popular statistical test used to evaluate the relationship between two variables [89]. Most of the time, the chi-square test is applied to test the dependence of the occurrence of the term and the occurrence of the class. Moreover, it is commonly used as a feature selection method. For example, the following formula is used to calculate the rank of terms that appear in the corpus:

(3) $$\begin{array}{c} \chi^{2}\left(D,t,c\right)=\underset{e_{t}\epsilon \left\{0,1\right\}}{\sum }\underset{e_{c}\epsilon \left\{0,1\right\}}{\sum }\frac{{\left(N_{e_{t}e_{c}}-E_{e_{t}e_{c}}\right)}^{2}}{E_{e_{t}e_{c}}}\text{.} \end{array}$$

Here, *e*_*t*_ and *e*_*c*_ are binary variables in the contingency table, *t* is the term, *c* is the class, *D* is the corpus, *N* is the observed frequency, and *E* is the expected frequency. The term *t* and class *c* are said to be dependent if *χ*^2^ is high. Thus, making term *t* an important feature that causes term *t* to indicate class *c*.

Table 8 shows important words within ISIS SEED, WS SEED, and MIWS datasets obtained by applying the chi-square test. Each dataset has a few repeated words. This can be attributed to different ideologies, sources, and dataset sizes.

### 2.8. Inferences

As seen from Table 6, cosine similarity proves that the obtained classes, namely, propaganda, radicalization, and recruitment are significantly different. The Wilcoxon signed-rank test also shows significant differences between the classes, so they have distinct features to make them unique. The chi-square test in Table 8 shows distinct word features to depict propaganda, radicalization, and recruitment.

Thus, it can be inferred that the newly formed classes propaganda, radicalization, and recruitment stand unique with statistical validation methods.

### 2.9. Dataset Evaluation

#### 2.9.1. Experimental Setup

Experiments were carried out on the HP Workstation Z8 G4 machine. It is equipped with a Xeon processor of 3 GHz, 128 GB of RAM, and Nvidia Quadro P400 GPU with 2 GB memory. In addition, some experiments were carried out on Nvidia DGX-Server with 4 Nvidia Tesla V-100 GPUs with 32 GB memory. Due to the limited capability of these systems, Google Colab was used. All the results in Table 9 are obtained on Google Colab.

#### 2.9.2. Size of Datasets

The size of datasets are provided in Table 10.

#### 2.9.3. Analyzing Imbalance in Datasets

The balance and imbalance in datasets are shown in Table 11.

### 2.10. Feature Extraction Techniques

To create word vectors, different feature extraction techniques are used in online extremism. In this work, the following feature extraction techniques are used:

#### 2.10.1. Unigram with TF-IDF

As seen in Table 9, the TF-IDF is used as the feature extraction technique. TF-IDF gives important words in the document based on its weightage in corpus [90]. Thus, TF-IDF was chosen, as it shows the word importance and is also used in many studies. Unigrams are considered to identify and elevate the importance of unique words representing the particular class, propaganda, radicalization, or recruitment.

#### 2.10.2. Bigrams and Trigrams with TF-IDF

Bigrams and trigrams features are used with TF-IDF for more complex analysis. These features provide the combination of words that affect the classification of the documents.

#### 2.10.3. Word2Vec

**Figure 14:**
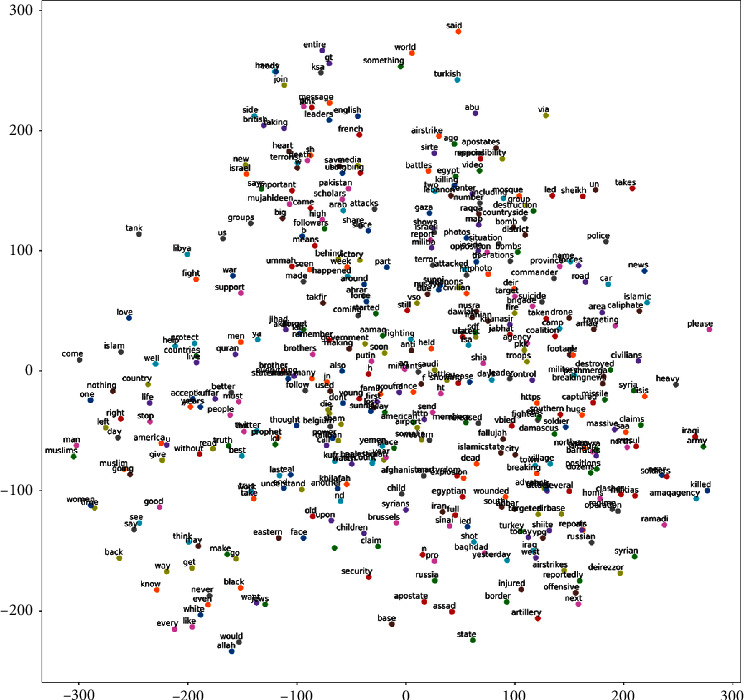
Word vectors using t-SNE and word2vec on MIWS dataset.

Word2vec uses a neural network to learn word embeddings or word vectors from the given corpus. Word2vec is used to gather more dimensional features to classify extremism text into propaganda, radicalization, and recruitment. The word2vec model pretrained on Google News with 300 dimensions was used for feature extraction in this work.  Figure 14 shows word vectors and their positions concerning each other using t-sne. Euclidean distance is used as a metric to calculate the distance between features. Thus, the lesser the Euclidean distance the more frequently the words appear together in a group. In Figure 14 it can be seen extremism influencing words are close to each other. Words such as “islamic state,” “dead,” “Afghanistan,” “wounded,” and “targeted” form a group. It can be also observed “bomb,” “raqqa,” “destruction,” “gaza,” “terror,” “attack,” and “battle” indicates the focus of groups on a particular location. The words such as “white,” “muslims,” “muslim,” and “black” stood out from other keywords indicating their usage in different contexts. Thus, word2vec can be effectively used for online extremism detection. Word2vec is used in combination with classifiers mentioned in the next section. Word2vec is fine-tuned to a window size of 15, a minimum count of 10 words, and with ten iterations to provide the best possible performance metrics.

### 2.11. Classifiers

To classify and predict, this work uses the following ML algorithms:

#### 2.11.1. Multinomial Naïve Bayes

MNB works on the probabilistic principle. Naïve Bayes assumes that there exists a conditional independence between every pair of features. In addition to this MNB, also assumes that distribution for all pair is multinomial distribution. This assumption of multinomial distribution works well in the case of word counts in the document. Thus, classifying text data based on the probabilistic appearance of a word within the document helps to get a baseline for performance metrics.

#### 2.11.2. Support Vector Machine

In online extremism detection, SVM can separate important words of a particular group or class by defining the exact separation line. This separation line is referred to as a hyperplane. SVM creates support vectors that are at the optimal distance from the hyperplane. This ensures the words of a particular group are at a significant distance from words of another group. So, one can get fairly accurate performance metrics due to this property of SVM.

#### 2.11.3. Random Forest

Random forest uses multiple decision trees to classify data. Every decision tree consists of decision nodes, root nodes, and leaf nodes. Thus, every decision tree in random forest is trained on a subsample of the dataset. Thus, each tree is ensured to be built upon the best subset of features. It takes the majority output of the decision trees to arrive at the classification. This reduces overfitting, thus making random forest a good choice for the extremism text classification.

#### 2.11.4. XGBoost

XGBoost uses gradient boosting for the classification. In XGBoost, gradient boosting is achieved by pruning trees backward that exceed the maximum depth of tree criteria, thus, increasing the speed of the algorithm by employing the depth-first technique. XGBoost can also work with a small amount of data. XGBoost also supports out-of-core computing, that is, it can handle data more than disk space and memory. Another advantage of XGBoost is, it provides parallelization, thus making the classification process faster.

**Figure 15:**
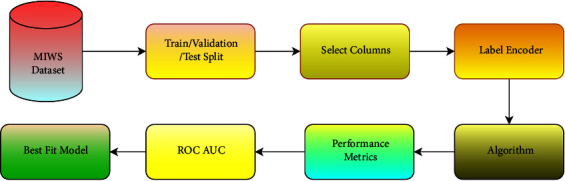
ML pipeline.

Figure 15, provides details about the ML pipeline for the best-fit model. In this pipeline, the MIWS dataset with preprocessed data is taken as input. Table 10 shows the count of tweets while Table 11 describes data imbalance in datasets used in this study. Then, train/validation/test split is performed on selected data. Different split ratios are used such as 60 : 20 : 20, 70 : 15 : 15,80 : 10 : 10, and 90 : 05 : 05. The better results were obtained for the 90 : 05 : 05 split. Particular columns such as preprocessed text and labels are selected for classification. As the labels are in string format, a label encoder is used to convert labels into the numerical format. Multiple ML algorithms as mentioned before are provided with GridSearchCV. The hyperparameters used for ML algorithms are shown in Table 12. The ML algorithms are scored on basis of performance metrics such as precision, recall, and F1 score. The ROC-AUC curve is also created for the visualising the performance of algorithms. On the basis of performance metrics and the ROC-AUC curve, the best-fit model is selected. A total of 64 experiments were conducted to get consistent results. The final models for every algorithm provided stable results as shown in Table 12. The bold values in Table 12 indicate the best results due to these hyperparameter values.

## 3. Results and Discussion

Multiple machine learning classifiers are used to assess and measure the classification performance of extremism data into *propaganda*, *radicalization,* and *recruitment*. The algorithms used are MNB, SVM, random forest, and XGBoost. These machine learning classifiers are chosen as they have been popularly used in online extremism detection research [36, 62].

### 3.1. Comparison of TF-IDF Unigram Results

**Figure 16:**
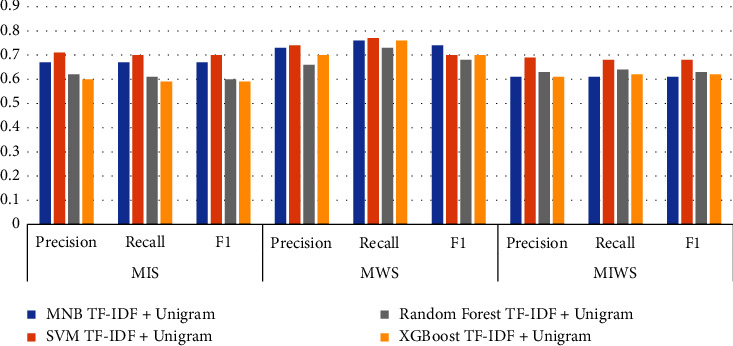
Performance metrics of algorithms on all datasets with TF-IDF unigram.

**Figure 17:**
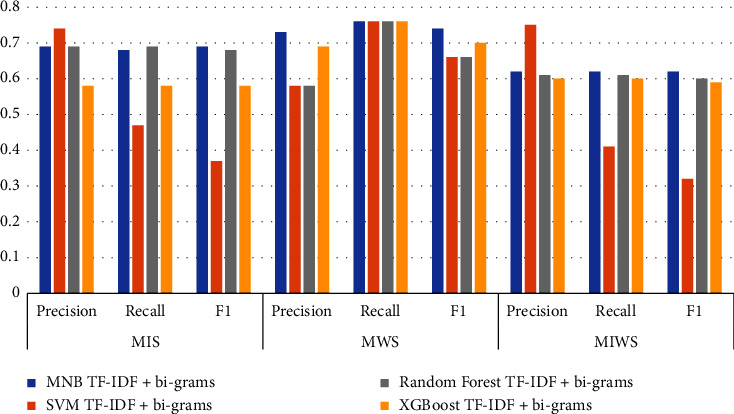
Performance metrics of algorithms on all datasets with TF-IDF bigrams.

**Figure 18:**
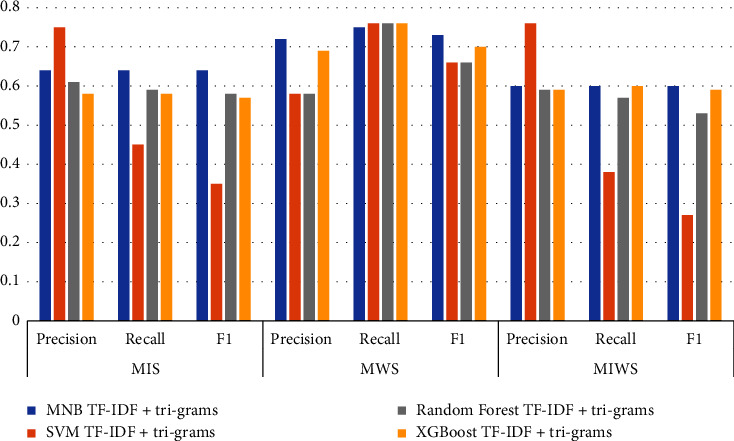
Performance metrics of algorithms on all datasets with TF-IDF trigrams.

**Figure 19:**
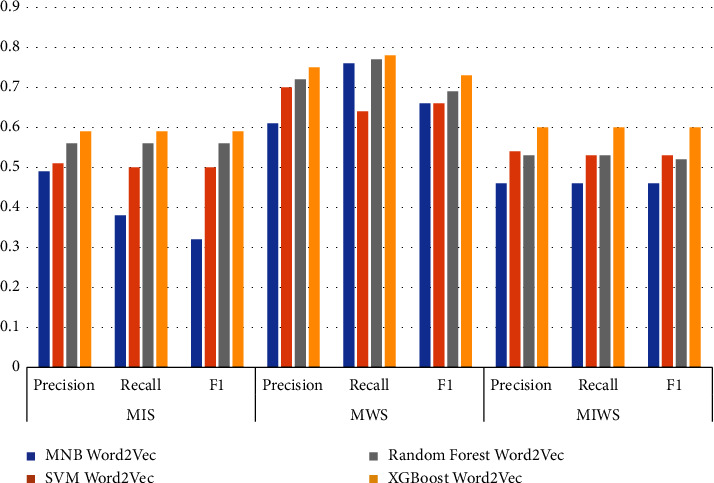
Performance metrics of algorithms on all datasets with word2vec.

Figures 16–19 shows the comparative performance of four feature extraction techniques with classifiers. It can be observed from the figures that TF-IDF unigram outperforms other feature extraction techniques, as unigram extracts the unique words that characterize the class. On the other hand, bigrams and trigrams offer comparatively low performance compared to unigrams for the frequent combinations of words in the multi-ideology MIWS dataset.

Word2vec with XGBoost offers comparable performance for the MIWS dataset, as it is pretrained on Google News data, as Google News may have accounted for extremism text. XGBoost with word2vec gives an F1 score of 0.60. It is also observed that word2vec can achieve better performance with more training epochs.

### 3.2. ROC-AUC (Unigram) for All Classifiers for MIWS

**Figure 20:**
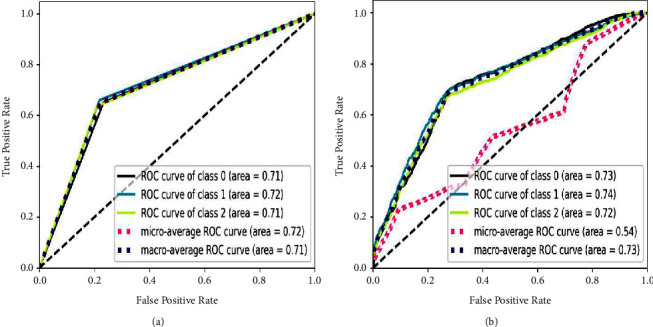
ROC curve for MIWS with TF-IDF unigram using (a) MNB and (b) SVM.

**Figure 21:**
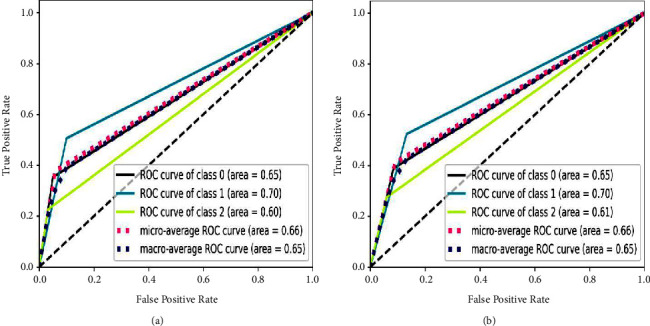
ROC curve for MIWS with TF-IDF unigram using (a) random forest and (b) XGBoost.

Receiver operating characteristics (ROC) is the graph that shows the performance of classification models at all classification thresholds [91]. Area under curve represents that the total two-dimensional are underneath ROC curve [92]. Figures 20 and 21 show the relative performance of chosen classifiers with the same feature extraction techniques, TF-IDF and unigram.

ROC-AUC are chosen for finding the relative performance of classifiers as they are focused on true positive values for multiclasses propaganda (Class 0), radicalization (Class 1), and recruitment (Class 2).

It is observed that the performance of all classifiers on the MIWS dataset is satisfactory, with an AUC of around 0.70 for MNB and SVM. For random forest and XGBoost, the AUC is around 0.65. Thus, it can be said that SVM with TF-IDF unigram outperforms other classifiers. Furthermore, SVM performs better due to marginalizing classes based on the unique words present in the MIWS dataset.

### 3.3. Multiclass Classification (Labelwise Precision and Recall and F1 Score with Support)

A total of 64 experiments were conducted to obtain consistent results across algorithms and features combined with different random states. Four experiments for each combination of algorithm and feature were carried out.  Table 13 provides the standard deviation of results on MIWS dataset. It can be observed that standard deviation is quite low. Thus, the results are stable.  Table 14 provides rank for the algorithm with features based on results in Table 9. Freidman rank test was performed to determine a rank-based significance for obtained results. The calculated p value by Freidman test was less than 0.05, that is, 1.7651*e* − 8. As seen in Tables 9 and 14, the ranks were calculated in descending order of results, so the lesser the rank, the more significant the results are. Therefore, SVM + TF-IDF results are significant and better than other algorithms and feature combinations.

Tables 15–18 give precision, recall, F1 score, and support for the TF-IDF unigram on the MIWS dataset for the chosen classifiers. It can be observed that SVM is the best classifier for propaganda, radicalization, and recruitment classes with an F1 score of 0.68, 0.72, and 0.63, respectively.

### 3.4. MIS Dataset

As seen in Table 9, SVM with TF-IDF unigram provides better results than MNB, random forest, and XGBoost for MIS dataset. For the MIS dataset containing jihadist-ISIS ideology, SVM with TF-IDF provides a better F1 score of 0.70. MNB and random forest with TF-IDF bigrams show an F1score of 0.69 and 0.68, respectively. MNB gives an F1 score of 0.64 for TF-IDF trigrams, exceeding other classifiers for the same feature. Only XGBoost shows better results using word2vec for feature extraction with an F1 score of 0.59.

### 3.5. MWS Dataset

For the MWS dataset, XGBoost with word2vec outperforms all other features extraction and classifiers used. Table 9 shows that XGBoost with word2vec gives a precision, recall, and F1 score of 0.75, 0.78, and 0.73, respectively. This can be attributed to the unique words in the MWS dataset, which may frequently appear in Google News data. For TF-IDF unigram, bigram, and trigram, MNB outperforms other classifiers with an F1score of 0.74, 0.74, and 0.73.

### 3.6. MIWS Dataset

For the unigram features chosen, machine learning classifiers offer a better performance. MNB, SVM, random forest, and XGBoost give an F1 score of 0.61, 0.68, 0.63, and 0.62, respectively, for unigram features. SVM provides maximum performance if F1 scores are compared. This can be attributed to common unique words for MIS and MWS.

**Figure 22:**
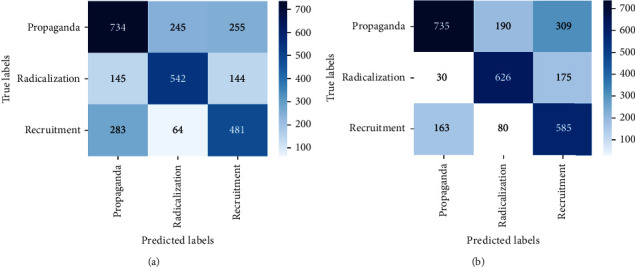
Confusion matrix of (a) MNB and (b) SVM.

**Figure 23:**
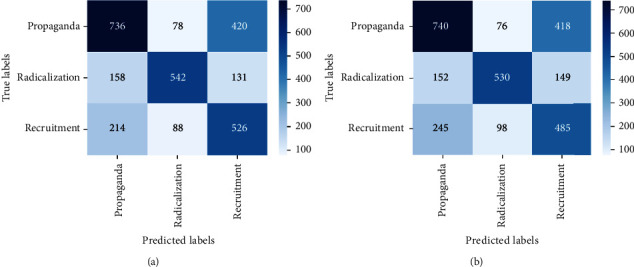
Confusion matrix of (a) random forest and (b) XGBoost.

For bigram and trigram features, the performance of algorithms reduces drastically. This can be attributed to different words based on the ideologies that are merged in a single dataset. Thus, bigram and trigram may not be effective in identifying and analyzing multiple ideologies together. Word2vec gives better performance for XGBoost. The F1score obtained from XGBoost with word2vec is 0.60. Figures 22 and 23 show the confusion matrix obtained by applying MNB, SVM, RF, and XGBoost on the MIWS dataset.

### 3.7. Inferences and Discussion

As seen in Tables 9–18, the results are a bit low. This is due to the merging of two different ideologies as the aim is to develop a generalized and ideology-independent extremism detection model. Methods and techniques to improve the results are discussed in the Section, Future Work.

Table 9 shows the comparative performance of the classifiers on the different feature extraction methods. The MIWS dataset with ∼17,000 ISIS and ∼11,000 WS examples is a multi-ideology dataset. The extremist dataset was developed and validated with three statistical methods that proved that the dataset is robust with the unique features in the three classes. The performance of ML algorithms on these extracted features in the dataset also shows potential for applying DL classifiers.

### 3.8. Limitations

The size of the dataset is an important aspect of machine learning. However, the size of the SEED dataset used in this work is limited, with fewer research articles. This is due to the lower availability of extremist text examples classified as propaganda, radicalization, and recruitment in the existing literature. Even with data imbalance, current data provides acceptable results, but balanced data is required to predict extremist text with precision.

The extremist text in the existing literature was manually labeled as propaganda, radicalization, and recruitment by experts. However, this labeling is limited by interrater agreement or expert opinion in the existing literature. Thus, the SEED dataset that is employed for topic modeling has the threat of expert bias. Hence the work relies on statistical validation techniques to verify the strength of the dataset. Furthermore, it is challenging both experimentally and ethically to quantify the bias of experts. Hence, at current stage of research it is not possible to compare the bias of both experts and the ML algorithm.

In this work, only three different topics or classes are considered for extremism classification text. Therefore, these topics were identified using simple LDA. The context-aware LDA [93] or context-aware topic modeling could be used to extract multiple different topics within extremism text.

Rigorous statistical tests were essential for estimating the strengths of the topic clusters. This work employed cosine similarity, Wilcoxon signed-rank, and chi-square tests for data validation as they were popularly employed in the literature. However, more statistical tests can be additionally employed to ensure the quality of data.

In this work, only four feature extraction techniques and four machine learning classifiers are employed on the developed MIWS dataset. Therefore, the results are limited by the choice of these representative classifiers and feature extractors. The classification and feature extraction purpose was to realize the model that would accurately classify the dataset.

A variety of advanced feature extraction techniques such as pretrained vectors can be further evaluated for a better accuracy. Advanced classifiers andtransformers can also be employed for achieving better accuracy.

## 4. Conclusion

This work focuses on constructing a multi-ideology and multiclass extremism text dataset with a comparative analysis of the performance of features extraction techniques and machine learning classifiers. Most extremism research studies focuses on a single ideology, with binary or tertiary classification such as extremist, nonextremist, and irrelevant classes. Consequently, there are limited insights from such works [19].

In this work, we develop a multi-ideology dataset with the most popular jihadist-ISIS and White supremacist ideologies. This dataset provides a broader view of extremism text with popular extremist ideologies brought together for better insights into data. The dataset also builds a multilabel extremist text dataset by classifying data as propaganda, radicalization, and recruitment.

The extremist text dataset was made contemporary by collecting extremist texts from different data sources (Twitter, ISIS Kaggle, StormFront dataset, and Gab dataset). In addition, we created ideology-specific datasets, which are called MIS (jihadist-ISIS), MWS (White supremacist), and proposed MIWS (multi-ideology) datasets with data preprocessing techniques applied.

A SEED dataset was created using existing literature that provided us with labeled examples of propaganda, radicalization, and recruitment. Then, the labeled SEED dataset was used to group/cluster the MIS, MWS, and MIWS datasets into propaganda, radicalization, and recruitment by using the LDA technique and cosine similarity. The grouping/clustering was further validated using statistical techniques. In this work, three different statistical tests, such as cosine similarity, Wilcoxon signed-rank test, and chi-square test, validated data labeling. Thus, our work is free from expert bias resulting due to manual validation such as previous literature. The visualization of word vectors with t-sne is also performed to highlight the unique words in propaganda, radicalization, and recruitment classes from the MIWS dataset.

To assess the performance of datasets, multiple features such as TF-IDF (unigram, bigram, and trigram) and pretrained word2vec (Google News) are used. These features were provided as input to classifiers such as MNB, SVM, RF, and XGBoost. For the proposed MIWS dataset, TF-IDF unigram with SVM provides the highest precision of 0.69, recall of 0.68, and F1score of 0.68. Thus, the results obtained using ML algorithms can be considered as a baseline for future work consisting of deep learning techniques.

This work, pioneers in developing the multi-ideology extremism text, MIWS dataset can classify extremism data into multiclasses such as propaganda, radicalization, and recruitment with robust statistical data validation techniques employed. Furthermore, this work investigates the best feature extraction technique and classifier for the proposed MIWS dataset, which guarantees better classification performance.

### 4.1. Future Work

The presented work is an important milestone in online extremism text detection research. This will open multiple avenues in the following research areas:

#### 4.1.1. Versatility of Extremism Text Dataset

Our work proves that multi-ideology datasets create a broader view of extremism text with comparable classification performance over single-ideology datasets. In the future, the presented dataset can be made more versatile with other popular extremist ideologies and sources. Increasing the SEED dataset also may produce more significant results. Different techniques such as word mover's distance [94] can also be used to calculate and improve the similarity between labels and topics.

#### 4.1.2. Feature Extraction Techniques

Context-aware topic modeling can be used to extract multiple different topics such as promoting violent acts and antisemitism. Popular feature extraction techniques such as pretrained vectors, GLoVe [95], and FastText [96], can be employed to extract complex relationships among extremism data. These can further enhance the accuracy of extremism detection models.

#### 4.1.3. Transfer Learning and Deep Learning Approaches

This research work uses machine learning classifiers for evaluating the proposed dataset. Future works can use deep learning models such as LSTM and CNN, and pretrained networks such as FastText, BERT, or RoBERTa for a better semantic analysis of extremism data. This can help achieve a higher performance for the classification of extremism text into propaganda, radicalization, and recruitment.

#### 4.1.4. Detection of Extremism Based on Geographical Context

The geographical location of extremists and extremist organizations plays an important role in analyzing propaganda, radicalization, and recruitment on social media platforms. The researchers have used the tweet location to identify extremist affiliations. It is necessary to identify the targeted nations through the extremist text which will speculate the activities of extremists. So, the extraction of geographical locations can play a major role in providing insights into extremist propaganda, radicalization, and recruitment tactics.

## Figures and Tables

**Table 1:** Datasets.

Dataset	Dataset type	Source	Language	Data collection period	Labels and percentage in dataset				Validation methods
ISIS Kaggle dataset [22]	Standard	Twitter	English	2015	No labels				No validation

About ISIS Kaggle dataset [37]	Standard	Twitter	English	2016	No labels				No validation

ISIS Religious Text Kaggle dataset [38]	Standard	Twitter	English	2014–2017	Quran			38%	No validation
					Hadith			27%	
					Other			35%	

StormFront [20]	Standard	Website	English	2017	Hate			11.29%	Manual validation: Cohen's kappa and Fleiss' kappa
					Nohate			86.09%	
					Relation			1.69%	
					Skip:			0.93%	

Gab Hate Corpus [21]	Standard	Gab	English	2016–2018	Assault on human dignity (HD)			8.5%	Manual validation: Fleiss' kappa and prevalence-adjusted and bias-adjusted Kappa (PABAK)
					Calls to violence (CV)			0.6%	
					Vulgar or offensive language (VO)			6.3%	

Berger and Morgan [25]	Custom	Twitter	Arabic, English, and French	2014	ISIS supporting accounts			93%	Manual validation

Chatfield et al. [16]	Custom	Twitter	English	2014	No labels				No validation

Rowe and Saif [39]	Custom	Twitter	English, Arabic, Dutch, and Spanish	Not specified	Pro-ISIS			0.4%	Manual validation: Fleiss' kappa

Kaati et al. [41]	Custom	Twitter	English and Arabic	2014	Pro-ISIS English			31.60%	No validation
					Random English			68.37%	
					Pro-ISIS Arabic			26.22%	
					Random Arabic			73.77%	

Benigni et al. [43]	Custom	Twitter	Not specified	2014	ISIS OEC member			15.38%	Manual validation

Abrar et al. [44]	Custom	Twitter	English	2018	Terrorism supporting			24.25%	No validation
					Terrorism nonsupporting			29.94%	
					Random			70.05%	

Ahmad et al. [45]	Custom	Twitter	English and Arabic	Not specified	Extremist			60.02%	No validation
					Nonextremist			39.79%	

Asif et al. [46]	Custom	Facebook	English and Urdu	2018	Moderate			27.07%	Survey-based validation
					High extreme			35.45%	
					Low extreme			15.34%	
					Neutral			22.13%	

Gialampoukidis et al. [47]	Custom	Twitter	English	Not specified	Not specified				No validation

Jaki and De Smedt [48]	Custom	Twitter	German and English	2017 F02D 2018	Hate			50%	No validation
					Safe			50%	

Berger [26]	Custom	Twitter	English, Spanish, and Dutch	Not specified	Not specified				Manual validation

De Smedt [49]	Custom	Twitter	English, Dutch, German, French, and Arabic	2014–2018	Jihadism			20%	No validation
					Extremism			40%	
					Racism			10%	
					Sexism			30%	

Berger [23]	Custom	Twitter	Not specified	2016	Nazi supporting accounts			50%	Manual validation
					ISIS supporting accounts	50%			

Heidarysafa et al. [50]	Custom	Magazines and website	English	2017	Not specified				Manual validation

Araque and Iglesias [36]	Custom	Twitter and magazines	English	2018–2020	Extremist: 50%				No validation
					Nonextremist: 50%				
					Hate: 58%				
					Nonhate: 42%				

**Table 2:** Popular machine learning techniques employed in online extremism detection.

Technique used for extremism detection	Study	Hyperparameter	Features	Performance metric	Remark
Naïve Bayes/multinomial Naïve Bayes	[35, 42, 46, 60–63]	Alpha = 0.01 [46]	n-grams, TF-IDF, and word2vec	Accuracy = 0.66 [46] and correctly classified instances = 89% [42]	Naïve Bayes or multinomial Naïve Bayes is used so as to build a model based on a probabilistic learning approach [46]

KNN	[64]	Distance = euclidean distance, *K* = 100 [64]	Term frequency	Precision = 0.48 and accuracy = 0.90 [64]	Distance-based approach for similarity in extremism text

Logistic regression	[36, 62, 65]	NA	Word2vec, fasttext, GloVe, and LIWC	F1 score = 99.77 [36] and accuracy = 0.70 [62]	Used for binary classification of extremism text

SVM	[36, 42, 44, 46, 49, 61–64, 66, 67]	Penalty = L1, tol = 1*e* − 3 [46]	n-grams, TF-IDF, word2vec, fasttext, GloVe, and PCA	Accuracy = 84 [67] and precision = 84 [49]	SVM segregates data using hyperplanes, so that classification is better. [46]

Random forest	[61, 63, 66, 68, 69]	Estimators = 100, Kfold = 5 [68], estimators = 100, max_depth = 50 [66]	n-grams, TF-IDF, word2vec, and GloVe	Accuracy = 100 [66] and F1-score = 0.93 [69]	Random forest is scalable and unaffected by outliers in extremism text dataset [66]

AdaBoost	[41, 42, 70]	Boosting iterations = 300 [41]	n-grams	Precision = 0.88 [70] and accuracy = 99.5 [42]	AdaBoost improves performance by combining weak classifiers

XGBoost	[59, 71]	Regularization = L2	Betweenness centrality and page rank	ROC-AUC curve = 0.95 [59]	XGBoost improves performance with faster learning

**Table 3:** Popular deep learning techniques employed in online extremism detection.

Technique used for extremism detection	Study	Hyperparameters	Features	Performance metric	Remark
CNN	[20, 73]	—	Embedding layer	Accuracy = 0.70 [20]	CNN 1D performs better for text classification [20]

LSTM	[20, 65, 73]	Layers = 4, units = 300, loss = binary cross entropy, optimizer = Adam, epochs = 10, batch size = 256 [65]	GloVe, Word2vec	F1 score = 0.7489 [65]	LSTMs are capable of handling long-term dependencies to learn efficiently from longer sentences

LSTM + CNN	[45]	LSTM: units = 100, max_features = 2000, and activation = “relu” CNN: kernel size = 2 × 2 [45]	Embedding layer	Accuracy = 92.68, and precision = 88.32 [45]	LSTM + CNN provides better results than only CNN and only LSTM

BERT	[65]	Layers = 24, units = 1024, learning rate = 2*e* − 5, epochs = 3, and batch size = 32 [65]	Word2vec, GloVe pre-trained on Twitter,	F1 score = 0.79605 [65]	BERT is used to understand the semantic context of words better and suited for the natural language queries

**Table 4:** Examples of propaganda, radicalization, and recruitment from the literature included in the SEED dataset.

Year	Study	Ideology	Propaganda	Radicalization	Recruitment
2015	Chatfield et al. [16]	Jihadist - ISIS	“Coalition planes massacred these children in airstrikes on #Hit, #Anbar”	“Himalaya888 you do realize IS wants to destroy every single nation-state, Arab or Kurd or communist does not matter, that they come across?”	“This is the time for muslim kurds in Turkey to show whether they can ever counter PKK.”

2015	Chatfield et al. [16]	Jihadist - ISIS	“These PKK fellas are exceptional liars. After the city was almost fully abandoned by civilians, they now claim 55 thousand civilians there.”	“Real warriors, I mean those who are not fags, dont need air support.”	“EbuRuana so if it is not right to make dua (the Islamic act of calling out to Allah) for a kafir (a disbeliever, someone who rejects Allah and who does not believe in Muhammad as the final messenger of Allah), what is the right thing to do ?”

2001	Ray and Marsh [77]	White supremacist	“Most victims of race crime, about 90 percent, are White. Almost 1 million White Americans were murdered, robbed, assaulted, or raped by black Americans in 1992, compared with about 132,000 blacks who were murdered, robbed, assaulted, or raped by Whites. Blacks are committing more than 50 times the number of violent racial crimes of Whites”	“He is someone who loves his race and his land more than he is concerned for his own welfare. He is neither an intellectual snob nor a street brawler. He is intelligent and educated enough to know what he is fighting for and brave enough to stand up for his beliefs when push comes to shove. He is someone who practices what he preaches, and who backs up his words with his deeds. He is definitely not someone who is long on talk and short on action.”	“(W)e need to take back America and the streets. I feel that Whites should be the predominant force in America. Whites can hardly walk down the street anywhere without being threatened. We must unite.”

2020	Johnson [11]	White supremacist	“America is dead! long live America!”	“Only right is right of lead”	“Join or die with rest”

**Table 5:** Examples from the Merged dataset.

Sr. no	Tweet	LDA topic	Ideology	Label
1	“Did not i tell you that JN only make takfir on those who spill their holy blood? SRF, hazm?”	0	Jihadist-ISIS	Propaganda
2	“Mujahideen from Burma capture 3 pigs of the Buddhist army”	1	Jihadist-ISIS	Radicalization
3	“Our prophet, has ordered us to fight you till you worship allah alone or give jizya”	2	Jihadist-ISIS	Recruitment
4	“They act as if negros with aids should have the right to reproduce when they will die off and have no way to take care of the kid I cannot believe that they are doing this by the millions its crazy”	0	White supremacist	Radicalization
5	“You are white and you are better than them and the next time they harass you and someone else form a group of buddies, go up to the principal office.”	1	White supremacist	Recruitment
6	“It is not right unless it is white.”	2	White supremacist	Propaganda

**Table 6:** Cosine similarity for classes.

Case	Classes	No. of examples (N1)	No. of examples (N2)	Cosine similarity	Inference
Case 1	Recruitment and propaganda	9,669	10,046	0.2857	Words representing classes are sufficiently dissimilar
		Recruitment	Propaganda		

Case 2	Radicalization and propaganda	13,937	10,046	0.29436	Words representing classes are sufficiently dissimilar
		Radicalization	Propaganda		

Case 3	Radicalization and recruitment	13,937	9,669	0.2993	Words representing classes are sufficiently dissimilar
		Radicalization	Recruitment		

**Table 7:** Cases, test statistics, and inferences.

Case	*n*	*W*	p value	Hypothesis testing	Inference
Case 1	19 and 325	46291754.5	1.023*e* − 18	Reject null hypothesis	Classes propaganda and recruitment differ considerably from each other
Case 2	19 and 325	51169402	6.181*e* − 64	Reject null hypothesis	Classes propaganda and radicalization differ considerably from each other
Case 3	19 and 325	61339810.5	2.441*e* − 16	Reject null hypothesis	Classes recruitment and radicalization differ considerably from each other

**Table 8:** Important words obtained by using chi-square.

Sr no	Propaganda			Radicalization			Recruitment		
	ISIS SEED	WS SEED	MIWS	ISIS SEED	WS SEED	MIWS	ISIS SEED	WS SEED	MIWS
1	Arab	Backwards	Aspect	Matter	Race	Killed	Bless	Adapt	Coalition
2	Massacre	Alien	Islamic	Call	Attack	Airstrikes	Counter	Accept	Martyrdom
3	People	Based	Beasts	Nation	Purity	State	Pkk	Stand	Behead
4	Assadis	Aboriginals	Allahu	Destroy	Scripture	Communist	Behead	Student	Adapt
5	America	Auschwitz	Apostate	Bullet	Call	Caliph	Believe	School	Munafiq
6	Apostate	Beasts	Assadis	Communist	Chosen	Amaqagency	Achieve	Friend	Believe
7	Possible	Aspect	Black	Single	Body	Race	Protect	Apologist	Iraqi
8	Babylon	Base	Israeli	Caliph	Believe	Nation	Place	Many	Accept
9	Back	Armed	Arab	State	Resist	Assault	Pledge	Antifa	Join
10	Photographer	Other	Operation	Realize	Revelation	Saudi	Point	White	Full

**Table 9:** Algorithms, features, and performance.

Sr no	Algorithm	Features	MIS			MWS			MIWS		
			Precision	Recall	F1score	Precision	Recall	F1 score	Precision	Recall	F1 score
1	MNB	TF-IDF	0.67	0.67	0.67	0.73	0.76	0.74	0.61	0.61	0.61
		TF-IDF + bigrams	0.69	0.68	0.69	0.73	0.76	0.74	0.62	0.62	0.62
		TF-IDF + trigrams	0.64	0.64	0.64	0.72	0.75	0.73	0.60	0.60	0.60
		Word2vec	0.49	0.38	0.32	0.61	0.76	0.66	0.46	0.46	0.46

2	SVM	TF-IDF	0.71	0.70	0.70	0.74	0.77	0.70	0.69	0.68	0.68
		TF-IDF + bigrams	0.74	0.47	0.37	0.58	0.76	0.66	0.75	0.41	0.32
		TF-IDF + trigrams	0.75	0.45	0.35	0.58	0.76	0.66	0.76	0.38	0.27
		Word2vec	0.51	0.50	0.50	0.70	0.64	0.66	0.54	0.53	0.53

3	Random forest	TF-IDF	0.62	0.61	0.60	0.66	0.73	0.68	0.63	0.64	0.63
		TF-IDF + bigrams	0.69	0.69	0.68	0.58	0.76	0.66	0.61	0.61	0.60
		TF-IDF + trigrams	0.61	0.59	0.58	0.58	0.76	0.66	0.59	0.57	0.53
		Word2vec	0.56	0.56	0.56	0.72	0.77	0.69	0.53	0.53	0.52

4	XGBoost	TF-IDF	0.60	0.59	0.59	0.70	0.76	0.70	0.61	0.62	0.62
		TF-IDF + bigrams	0.58	0.58	0.58	0.69	0.76	0.70	0.60	0.60	0.59
		TF-IDF + trigrams	0.58	0.58	0.57	0.69	0.76	0.70	0.59	0.60	0.59
		Word2vec	0.59	0.59	0.59	0.75	0.78	0.73	0.60	0.60	0.60

**Table 10:** Size of datasets.

Datasets	Source	Ideology	Total tweets/posts
Twitter tweets	Twitter	White supremacist	2,000
Twitter tweets	Twitter	Jihadist-ISIS	2,000
ISIS Kaggle dataset [22]	Twitter	Jihadist-ISIS	∼15,000
StormFront [20] + Gab dataset [21]	StormFront and Gab	White supremacist	∼9000 (only hate class)

**Table 11:** Balance and imbalance in datasets.

Datasets	Number of classes	Class 1	Class 2	Class 3
ISIS Kaggle dataset	1	15,438	—	—
StormFront dataset	2	1180 (hate)	8,537 (nohate)	—
Gab dataset	2	8,327 (hate)	∼25,000 (other)	—
MIS	3	7,214 (propaganda)	5,103 (radicalization)	51,21 (recruitment)
MWS	3	5,131 (propaganda)	3,214 (radicalization)	3,162 (recruitment)
MIWS	3	12,345 (propaganda)	8,317 (radicalization)	8,283 (recruitment)

**Table 12:** Hyperparameters used for fine-tuning ML algorithms.

Algorithm	CV	Learning rate/gamma/alpha	C (max depth)	Kernel (max_features)	n_estimators
MNB	10	0.1, 0.5, and **1**	—	—	—
SVM	10	1*e* − 3, 1*e* − 4, and **1e** − **5**	1, 2, and **3**	linear, poly, and **rbf**	
Random forest	10	—	300, **350**, and 400	**auto** and sqrt	**80**, 90, and 100
XGBoost	10	**0.1**, 0.01, and 0.001	4, **5**, and 6	—	500, **550**, and 600

Bold values are the optimal hyperparameters for the respective algorithm.

**Table 13:** Standard deviation of results for the MIWS dataset.

Algorithm	Features	MIWS		
		Precision	Recall	F1 score
MNB	TF-IDF	0.005774	0.011547	0.005774
	TF-IDF + bigrams	0.009574	0.005	0.008165
	TF-IDF + trigrams	0.01	0.01291	0.009574
	Word2vec	0.015	0.02	0.014142

SVM	TF-IDF	0.009574	0.01	0.005
	TF-IDF + bigrams	0.070711	0.025	0.017321
	TF-IDF + trigrams	0	0.01291	0.021602
	Word2vec	0.005	0.01	0.005

Random forest	TF-IDF	0.01	0.01	0.005
	TF-IDF + bigrams	0.005	0.005	0.005
	TF-IDF + trigrams	0.02	0.015	0.04
	Word2vec	0.005	0.005	0.005

XGBoost	TF-IDF	0	0.005	0.01
	TF-IDF + bigrams	0.009574	0.005	0.01893
	TF-IDF + trigrams	0.005	0.01	0.005
	Word2vec	0.008165	0.009574	0.008165

**Table 14:** Significant results by ranks.

Algorithm	Features	Rank
MNB	TF-IDF	4.5
MNB	TF-IDF + bigrams	2.5
SVM	TF-IDF	1
Random forest	TF-IDF	2.5
XGBoost	TF-IDF	4.5

**Table 15:** Label wise performance metrics for MNB TF-IDF.

Algorithm and feature	Class	Precision	Recall	F1 score	Support
MNB and TF-IDF	Propaganda	0.59	0.63	0.61	1234
	Radicalization	0.65	0.64	0.65	831
	Recruitment	0.58	0.55	0.56	828

**Table 16:** Label wise performance metrics for SVM TF-IDF.

Algorithm and feature	Class	Precision	Recall	F1 score	Support
SVM and TF-IDF	Propaganda	0.60	0.79	0.68	1234
	Radicalization	0.75	0.69	0.72	831
	Recruitment	0.71	0.56	0.63	828

**Table 17:** Labelwise performance metrics for random forest TF-IDF.

Algorithm and feature	Class	Precision	Recall	F1 score	Support
Random forest and TF-IDF	Propaganda	0.60	0.66	0.63	1234
	Radicalization	0.65	0.76	0.70	831
	Recruitment	0.64	0.49	0.55	828

**Table 18:** Labelwise performance metrics for XGBoost TF-IDF.

Algorithm and feature	Class	Precision	Recall	F1 score	Support
XGBoost and TF-IDF	Propaganda	0.60	0.63	0.62	1234
	Radicalization	0.64	0.75	0.69	831
	Recruitment	0.59	0.46	0.51	828

## Data Availability

The data used to support the findings of this study are included within the article.
